# Interferon-Tau Exerts Direct Prosurvival and Antiapoptotic Actions in Luteinized Bovine Granulosa Cells

**DOI:** 10.1038/s41598-019-51152-6

**Published:** 2019-10-11

**Authors:** Raghavendra Basavaraja, Senasige Thilina Madusanka, Jessica N. Drum, Ketan Shrestha, Svetlana Farberov, Milo C. Wiltbank, Roberto Sartori, Rina Meidan

**Affiliations:** 1 Department of Animal Sciences, The Robert H. Smith Faculty of Agriculture, Food and Environment, The Hebrew University of Jerusalem, Rehovot, Israel; 2 Department of Animal Science, University of São Paulo, Piracicaba, Brazil; 3 Department of Dairy Science, University of Wisconsin-Madison, Madison, WI United States

**Keywords:** Apoptosis, Endocrinology

## Abstract

Interferon-tau (IFNT), serves as a signal to maintain the corpus luteum (CL) during early pregnancy in domestic ruminants. We investigated here whether IFNT directly affects the function of luteinized bovine granulosa cells (LGCs), a model for large-luteal cells. Recombinant ovine IFNT (roIFNT) induced the IFN-stimulated genes (ISGs; *MX2*, *ISG15*, and *OAS1Y*). IFNT induced a rapid and transient (15–45 min) phosphorylation of STAT1, while total STAT1 protein was higher only after 24 h. IFNT treatment elevated viable LGCs numbers and decreased dead/apoptotic cell counts. Consistent with these effects on cell viability, IFNT upregulated cell survival proteins (MCL1, BCL-xL, and XIAP) and reduced the levels of gamma-H2AX, cleaved caspase-3, and thrombospondin-2 (THBS2) implicated in apoptosis. Notably, IFNT reversed the actions of THBS1 on cell viability, XIAP, and cleaved caspase-3. Furthermore, roIFNT stimulated proangiogenic genes, including *FGF2*, *PDGFB*, and *PDGFAR*. Corroborating the *in vitro* observations, CL collected from day 18 pregnant cows comprised higher ISGs together with elevated FGF2, *PDGFB*, and *XIAP*, compared with CL derived from day 18 cyclic cows. This study reveals that IFNT activates diverse pathways in LGCs, promoting survival and blood vessel stabilization while suppressing cell death signals. These mechanisms might contribute to CL maintenance during early pregnancy.

## Introduction

The corpus luteum (CL) plays a crucial role in the regulation of the reproductive cycle, fertility, and maintenance of pregnancy^1^. Progesterone secreted by the CL regulates uterine receptivity and promotes elongation, implantation, and survival of the growing embryo^2–6^. In a non-fertile cycle, prostaglandin F2alpha (PGF2a) secreted from the uterus, causes CL regression and terminates its progesterone production^1,7^. Our previous studies have highlighted the role of thrombospondins (THBSs) during luteolysis, both THBS1 and THBS2 were up-regulated by PGF2a administration, specifically, in mature, PGF2a-responsive CL but not during the refractory stage (before day 5 of the cycle)^8^. PGF2a also elevated THBS1 and THBS2 expression *in vitro* in luteal cell types^8,9^. Thrombospondins comprise a family of five conserved multidomain, large glycoproteins of which THBS1 and THBS2, are highly homologous, exhibiting similar structural and functional domains^10–12^.

Interferon tau (IFNT) is secreted from the mononuclear trophectoderm of the conceptus during the peri-implantation period and acts as the key signal for extending CL function during early pregnancy in domestic ruminants, a process known as maternal recognition of pregnancy (MRP)^13–15^. Consistent with this idea, administration of IFNT into the sheep uterine lumen or the systemic circulation during MRP extended the CL lifespan^16–18^. IFNT belongs to the type 1 IFN family of peptides, binds type-1 interferon cell-surface receptors (IFNAR1 and IFNAR2), and activates Janus kinase–signal transducer and activator of transcription–interferon regulatory factor (JAK–STAT–IRF) signaling resulting in the induction of a large number of classical interferon-stimulated genes (ISGs)^19–21^. The primary distinguishing feature between IFNT and most other type I IFNs is its unique, trophoblast-specific expression pattern ruminants. In cows, IFNT is secreted by the trophoblastic cells throughout days 10–24 of pregnancy^14,22^. The presence of elevated levels of ISGs in CL of pregnant animals suggested extra-uterine, endocrine effects of IFNT^16,18,23,24^. In agreement with the model of an endocrine action of IFNT acting directly on the CL during early pregnancy, we have previously demonstrated that recombinant ovine IFNT (roIFNT) enhanced luteal endothelial cell proliferation and suppressed a battery of luteolytic genes in luteal endothelial cells and CL slices^25^. The CL is a transient endocrine gland that contains multiple cell populations in which two progesterone-producing cells exist—large and small luteal cells^1,26,27^. Although the large luteal cells represent a minority of the luteal cell numbers, they are the cell type with the greatest luteal volume and are responsible for the majority of progesterone output from the CL^27–29^. Furthermore, unlike small luteal cells, large luteal cells produced progesterone in a LH-independent manner^29–31^, consistent with a key role for this cell type during pregnancy when circulating progesterone is elevated and GnRH and LH pulse frequencies are decreased^32^.

This study investigated the direct actions of IFNT on large luteal cells, utilizing luteinized granulosa cells (LGCs) as an *in vitro* model. In addition, we evaluated here CL of early pregnant cows (on day 18) to complement the *in vitro* data.

## Results

### IFNT induces STAT1 phosphorylation and ISGs in LGCs

**Figure 1:**
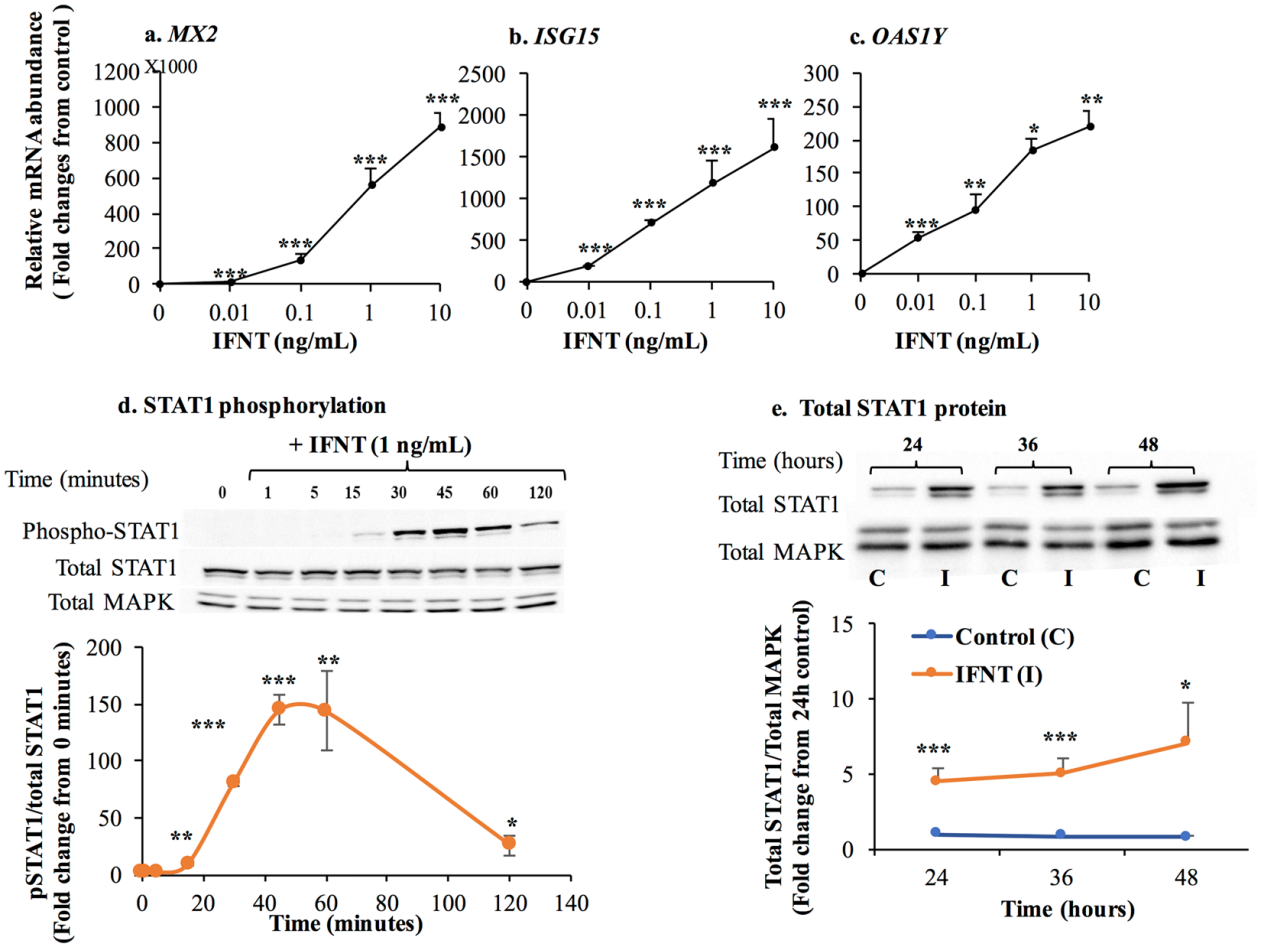
IFNT stimulated STAT1 dependent type-1 interferon pathway in LGCs. (**a**–**c**) Cells were incubated for 24 h with either basal media (control) or varying concentrations of roIFNT (0.01–10 ng/mL). Cells were then harvested, and the mRNA expression of (A) MX2, (**b**) ISG15 and (C) OAS1Y were determined using qPCR. (**d**) Tyrosine phosphorylated STAT1 (p-STAT1) and (**e**) total STAT1 proteins were analyzed using specific antibodies in western blotting. Representative images of western blots are presented in the upper panels of D and E. Blots in (**d**) were cropped from different gels, blots in (**e**) were cropped from different parts of the same gels. Whole cell extracts from LGCs were incubated with IFNT (1 ng/mL) for different time points as indicated. Quantitative analysis of phospho-STAT1 levels were normalized to total STAT1 and total STAT1 was normalized to total MAPK (p44/42) protein. Asterisks indicate significant differences from either time 0 or basal media (control) or control (*P < 0.05, **P < 0.01, ***P < 0.001).

In this study, cells were incubated with various concentrations of roIFNT (0.01–10 ng/mL), and the expression of classical ISGs was assessed to investigate whether IFNT directly affects LGCs. roIFNT dose-dependently, robustly, and markedly elevated the mRNA concentrations of *MX2*, *ISG15*, and *OAS1Y* in LGCs (Fig. 1a–c). Among these ISGs, *MX2* exhibited the highest fold induction in response to IFNT, followed by *ISG15* and *OAS1Y* (Fig. 1a–c). In addition, IFNT enhanced the STAT1 phosphorylation (Fig. 1d), at tyrosine 701 already 15 min after treatment. The greatest STAT1 phosphorylation was observed at 45 min after IFNT treatment with declining phosphorylation thereafter (Fig. 1d). The content of the total STAT1 protein remained unchanged during the short-term incubation (~2 h) with IFNT (Fig. 1d); however, total STAT1 protein increased during the prolonged incubation time (24–48 h; Fig. 1e). Furthermore, these cells readily expressed interferon-alpha receptor 1 (*IFNAR1*; data not shown).

### IFNT enhances LGCs survival and positively regulates proangiogenic factors

**Figure 2:**
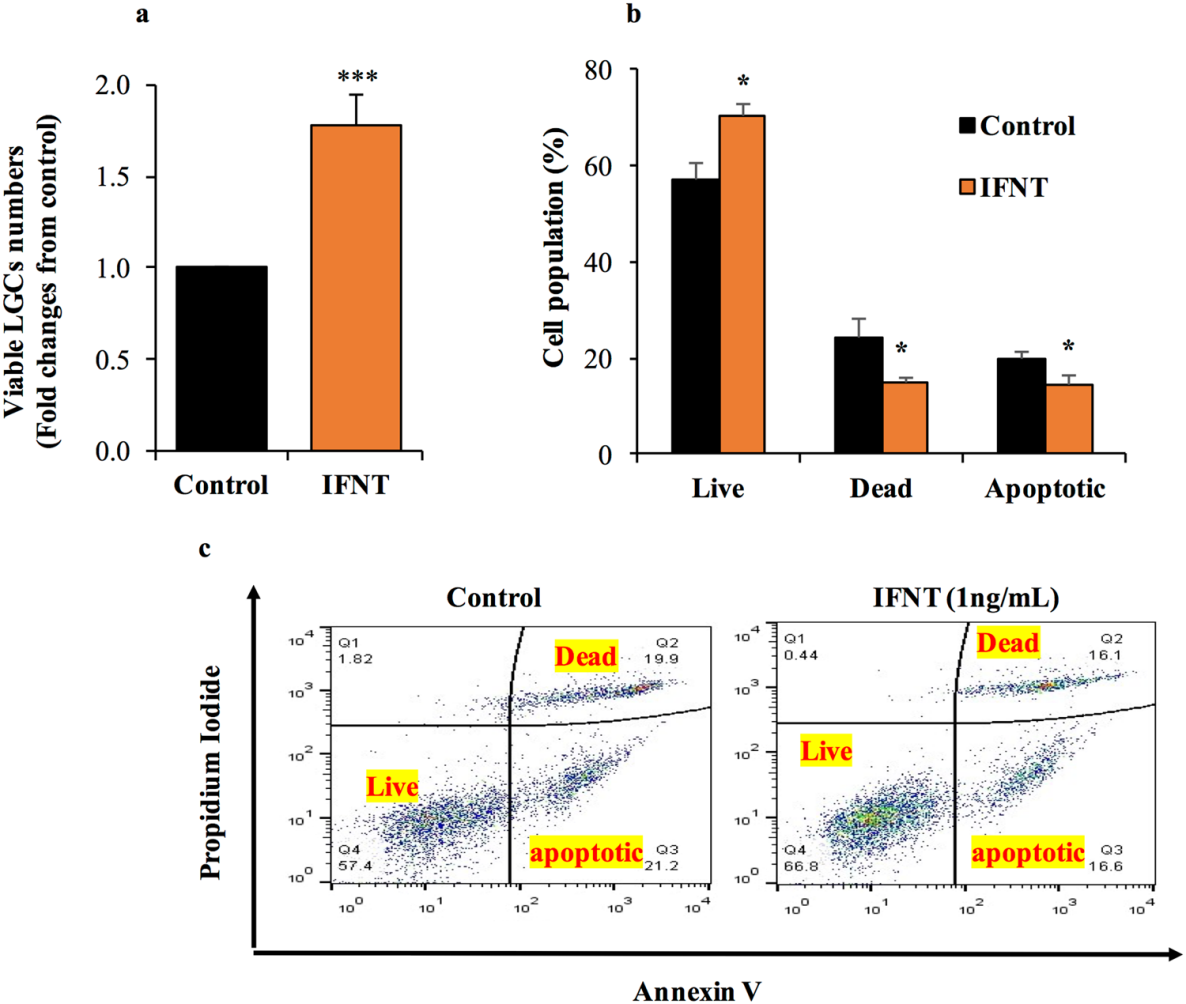
IFNT increased LGCs viability and reduced apoptosis. (**a**) Cells were assayed with XTT 48 h after treatment with 1 ng/mL of roIFNT. (**b**) Cells were treated with 1 ng/mL of roIFNT and analyzed using FACS with propidium iodide and Annexin V staining (representative plots). (**c**) Quantification of FACS results. Asterisk indicate significant (*P < 0.05) difference from cells treated in basal media (control).

Treatment with roIFNT (1 ng/mL) doubled viable LGCs numbers compared to the control (basal medium) based on the XTT assay (see Materials and Methods); Fig. 2a). Next, cells were stained with Annexin V–FITC and propidium iodide (PI) and were analyzed by flow cytometry. LGCs incubated for 48 h with 1 ng/mL of roIFNT (Fig. 2b,c) resulted in significantly fewer apoptotic and dead cells and greater numbers of live cells compared to control LGCs (Fig. 2b,c).

**Figure 3:**
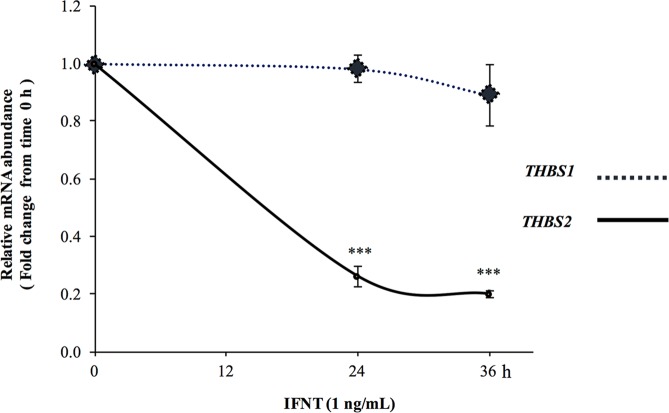
The effect of IFNT on THBSs mRNA in LGCS. Cells were incubated with roIFNT (1 ng/mL) for 24 and 36 h. Cells were then harvested, and the mRNA expression was determined using qPCR. Asterisks indicate significant differences from time 0 (***P < 0.001).

**Figure 4:**
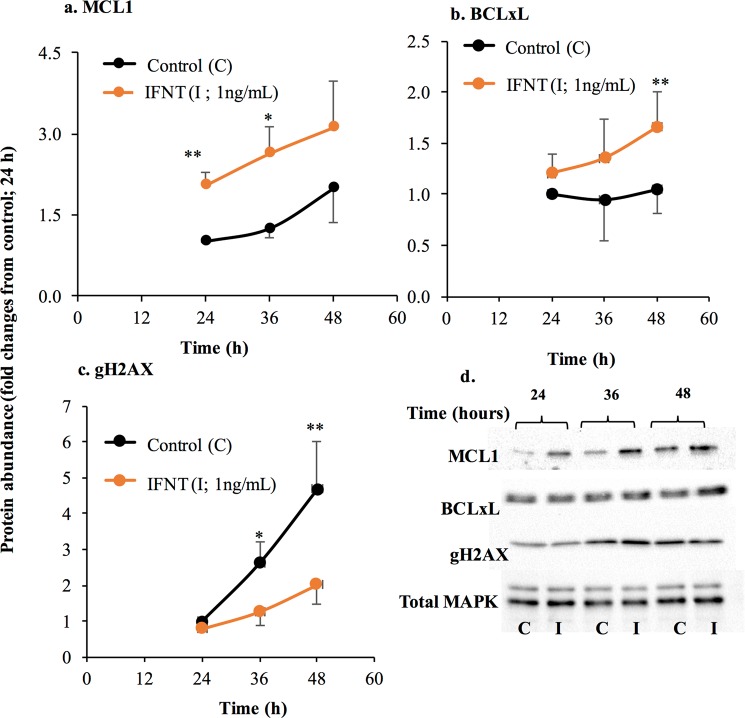
Time-dependent effects of IFNT on pro- and antiapoptotic proteins. LGCs were incubated without (control) or with roIFNT (1 ng/mL) for 48 h, (**a**) MCL1, (**b**) BCL-xL, and (**c**) gH2AX protein levels were determined in cell extracts by western blotting and normalized relative to the abundance of total MAPK (p44/42). All blots expect MCL1 were cropped from different parts of the same gels. Results are presented as the means ± SEM from four independent experiments. (**d**) Representative images of the western blots for each antibody. Asterisks indicate significant differences from their respective controls (*P < 0.05, **P < 0.01, ***P < 0.001).

**Figure 5:**
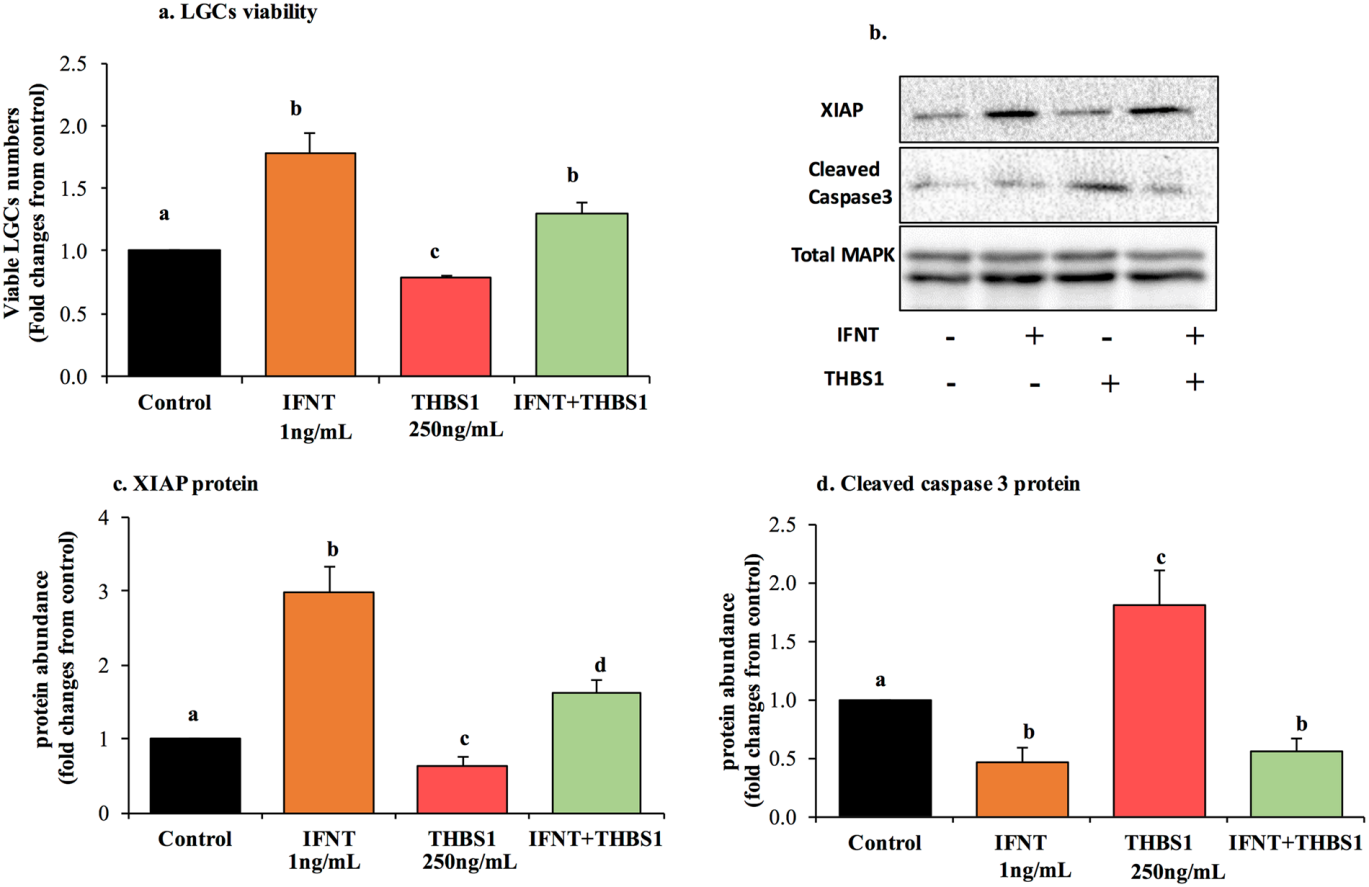
IFNT induces cell survival and counteracts THBS1 apoptotic actions. LGCs were treated with either with basal media (control), roIFNT (1 ng/mL), human recombinant THBS1 (250 ng/mL), or the combination of IFNT and THBS1 for 48 h. (**a**) The viable cell number, (**b**) Representative images of the western blots for each antibody, (**c**) XIAP and (**d**) cleaved caspase-3 proteins. Protein levels were determined in cell extracts by western blotting using specific antibodies and normalized relative to the abundance of total MAPK (p44/42). The results represent means ± SEM of four independent experiments. Asterisks indicate significant differences from their respective controls (*P < 0.05, **P < 0.01, ***P < 0.001).

Besides IFNT-mediated antiapoptotic effects, we noted a time-dependent decline in proapoptotic THBS2 mRNA expression^8,33^, which was sharply reduced by treatment with roIFNT at 24 h remaining low at 36 h after treatment without altering *THBS1* mRNA (Fig. 3). In addition, we assessed the effects of roIFNT on several proteins including myeloid cell leukemia 1 (MCL1) and B-cell lymphoma-extra-large (BCL-xL; anti apoptotic members of the BCL2 family), as well as the proapoptotic protein gamma H2A histone family, member X (gH2AX; Fig. 4a–c). There was a distinct temporal profile of induction for these proteins by IFNT. Treatment with roIFNT increased MCL1 within 24 h with a tendency for increased MCL1 protein at 36 h and numerically greater protein at 48 h. In contrast, there were no differences in BCLxL protein at 24 and 36 h after roIFNT treatment but there was a significant increase in compared with control (P < 0.01) at 48 h after roIFNT treatment. Compared with these two, gH2AX was inhibited by roIFNT (Fig. 4c) 36 and 48 h after treatment. In addition, we observed a marked treatment by time interaction for the gH2AX protein. Subsequently, we assessed whether roIFNT can alter apoptotic activities of THBS1described previously^9,34,35^. Treatment with THBS1 (250 ng/mL) reduced viable LGCs numbers (Fig. 5), corroborating previous results^9^. Moreover, treatment with roIFNT alone doubled viable LGCs numbers and decreased THBS1-mediated reduction in LGCs viability (Fig. 5a). These effects were also reflected in measuring X-linked inhibitor of apoptosis protein (XIAP); THBS1 decreased XIAP protein levels, while roIFNT markedly increased its basal levels and those suppressed by THBS1 (Fig. 5c). Figure 5d presents additional evidence for the opposing effects of IFNT and THBS1. Levels of cleaved caspase-3 protein, which is a protein that is activated in the apoptotic cell, were significantly decreased by roIFNT and increased in THBS1-treated cells. In addition, treatment with THBS1 combined with roIFNT resulted in amounts of cleaved caspase 3 protein that were not different from roIFNT-treated cells but lower than either control or THBS1-treated cells (Fig. 5d).

**Figure 6:**
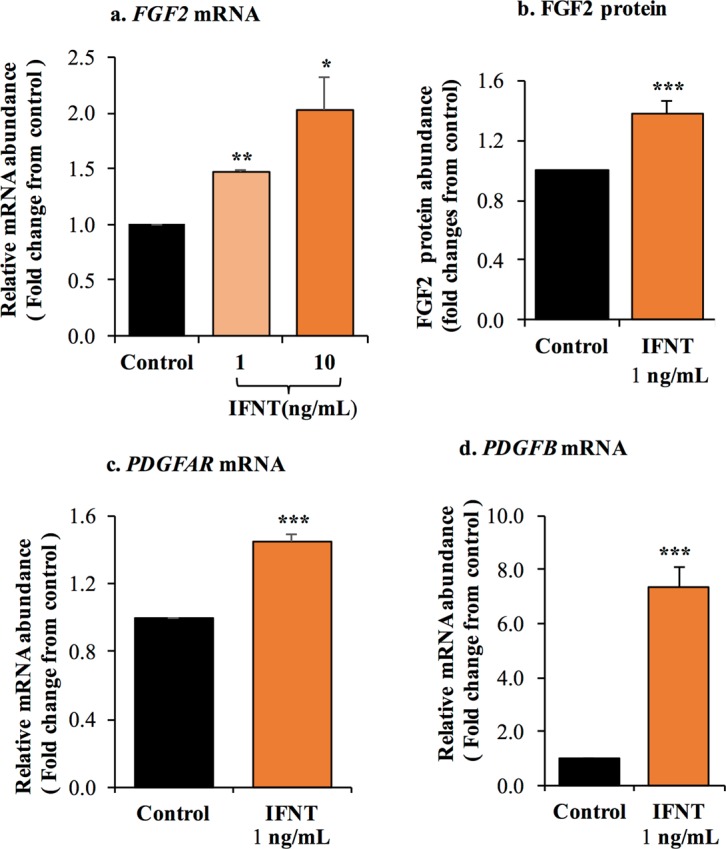
IFNT promotes proangiogenic factors in LGCs. LGCs were treated with either basal media (control) or roIFNT (1 and 10 ng/mL). (**a**,**b**) FGF2 mRNA and protein levels. (**c**) *PDGFB* and (**d**) *PDGFAR* mRNA expression. Asterisks indicate significant differences between roIFNT treatment and controls (*P < 0.05, **P < 0.01, ***P < 0.001).

Next, we evaluated the effect of IFNT on several proangiogenic factors expressed in LGCs, there was an increase of *FGF2* (fibroblast growth factor-2) mRNA (Fig. 6a) and protein (Fig. 6b) in response to roIFNT. Treatment with roIFNT also markedly elevated mRNA concentrations for *PDGFB* and its receptor *PDGFAR* (Fig. 6c,d).

### Comparison of gene expression in CL collected on day 18 CL from pregnant and non-pregnant cows

**Figure 7:**
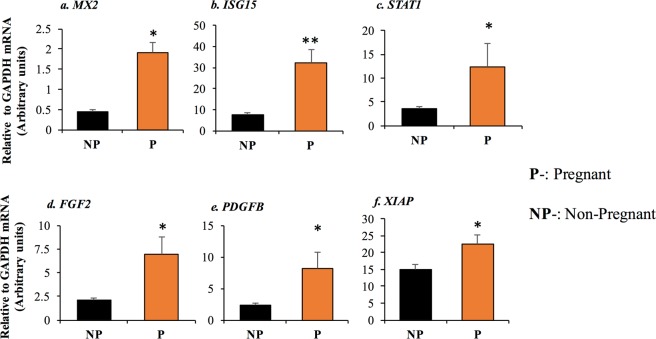
Differential gene expression in pregnant and cyclic CL. The levels of (**a**) *ISG15*, (**b**) *MX2*, (**c**) *STAT1*, (**d**) *FGF2*, (**e**) *PDGFB*, and (**f**) *XIAP* mRNAs in day 18 CL from pregnant (P) and cyclic (C) cows. The total RNA was isolated from the tissue, and the mRNA expression were determined using qPCR. Asterisks indicate significant differences between pregnant and cyclic CL (*P < 0.05, **P < 0.01, ***P < 0.001).

To determine whether genes that were stimulated *in vitro* by roIFNT were also upregulated during early pregnancy *in vivo*, CL were collected from pregnant cows on day 18 after AI, when IFNT concentrations are expected to be elevated^14,22^ or in cyclic non-inseminated cows on day 18 of the estrous cycle. Plasma progesterone was not different between pregnant (5.97 ± 0.79 ng/ml, n = 6) and cyclic (5.81 ± 1.10 ng/ml, n = 6) cows, confirming that CL were not undergoing regression at the time of collection. Figure 7a–c shows that day 18 pregnant CL exhibited greater mRNAs levels of *MX2* (4.3-fold), *ISG15* (4.2-fold), and *STAT1* (3.3-fold) compared with non-pregnant cows on day 18, implying that IFNT was indeed elevated in pregnant animals. Besides elevated ISGs, *FGF2* (3.4-fold), *PDGFB* (3.5-fold), and *XIAP* (1.5-fold) mRNAs were also higher in the CL derived from pregnant cows compared with day 18 cyclic cows (Fig. 7d–f).

## Discussion

The evidence provided by this study portray IFNT as an important survival factor for the large luteal-like cell. Treatment with IFNT increased viable LGCs numbers, reduced apoptotic cells, and increased prosurvival proteins such as XIAP, MCL1, and BCL-xL, while inhibiting antiapoptotic proteins, active caspase-3, and gH2AX. Notably, IFNT could also rescue THBS1-mediated apoptotic actions in LGCs. Concurrently with its prosurvival effects, IFNT stimulated proangiogenic factors –FGF2, PDGFB, and its receptor –PDGFAR. Although *in vitro* luteinized granulosa cells were repeatedly shown to be a reliable model for the bovine large luteal cells^36–40^, future studies employing CL -derived cells may ascertain these mechanisms in the early pregnant CL. In fact, CL derived from day 18 pregnant cows had elevated ISGs levels, along with higher XIAP, FGF2, and PDGFB compared with cyclic cows on day18, corroborating the *in vitro* data.

In domestic ruminants, IFNT initiates pregnancy recognition signaling, maintaining a favorable uterine environment for the successful establishment of pregnancy^41^. It is well defined that the primary antiluteolytic actions of IFNT are exerted on the endometrium to prevent the pulsatile release of the PGF2a^42–45^. Nevertheless, IFNT has also been detected in the uterine vein serum of ewes on days 15 and 16 of pregnancy, suggesting its presence in circulation^17,46^. Indeed, extra-uterine or endocrine effects of IFNT have been reported in domestic ruminants. Moreover, elevated ISGs were observed in peripheral blood mononuclear cells, liver, mammary glands, lymphatic cells, and CL during early pregnancy^16,24,47–49^. Of these tissues, the CL occupies central significance because of its roles in establishing and maintenance of early pregnancy. More than the stimulation of ISGs, IFNT increased *in vitro* lymphatic bovine endothelial cell proliferation and capillary-like tube formation, suggesting its involvement in luteal lymph angiogenesis^48^. Recently it was reported that, IFNT increases IL8 in bovine luteal cells, suggesting that it can change immune microenvironment in the CL^49^. Furthermore, we have previously showed that IFNT activates the STAT1-dependent type-1 interferon pathway and stimulates ISGs in bovine luteal endothelial cells and CL slices^25^. The present study further supports this physiological concept, utilizing LGCs, we show that IFNT induces temporal STAT1 phosphorylation and the expression ISGs. Elevated ISGs were also observed in this study in CL of day 18 pregnant cows. Together, these observations provide strong evidence for IFNT responsiveness in CL and in luteal cells and for IFNT action in the CL during early pregnancy.

Apoptosis is convincingly implicated as a mechanism triggering CL demise in ruminant species^50–55^. Conversely, CL maintenance requires the suppression of apoptotic mechanisms and augmentation of survival signals^55^. Thus, a delicate balance between survival and apoptotic factors determine luteal cell fate. The BCL2 family proteins comprise a network that regulates intrinsic survival/apoptotic responses^56^ and contains anti- and proapoptotic proteins. There are BCL2 homologs that promote cell survival such as BCL2, BCxL, and MCL1. The proapoptotic BCL2 family members^57^ contain BH1 to BH4 domains which interact with antiapoptotic BCL2 homologs, preventing mitochondrial outer membrane permeabilization. XIAP is another major antiapoptotitc protein, which can directly bind and inhibit caspase 9 and the active effector protein caspase-3^58,59^. BCL-xL and XIAP proteins are elevated in the sheep CL on days 12–16 of pregnancy compared with cyclic CL on the same days^55^. Likewise, we observed in this study that day 18 pregnant bovine CL had higher levels of *XIAP* than cyclic cows. Furthermore, the endocrine delivery of IFNT to cyclic ewes on day 10 was also found to elevate the mRNA levels of cell survival genes, such as *BCL-xL* and *XIAP*, compared with ewes treated with a vehicle only^23^. Thus, findings of this study demonstrating that IFNT enhanced the levels of MCL1, BCL-xL, and XIAP proteins but decreased cleaved caspase-3, are physiologically meaningful. Another critical apoptotic marker that was effectively suppressed by IFNT was gH2AX, a phosphorylated form of histone H2AX that can function as a sensitive marker for double-strand breaks, and it is highly correlated with the induction of apoptosis^60,61^.

This study also provides evidence demonstrating the ability of IFNT to reverse the known apoptotic actions of THBS1 on viable LGCs numbers, XIAP, and cleaved caspase-3. Previously we reported that IFNT downregulated THBS1 in bovine CL slices and luteal endothelial cells *in vitro*^25^. This study shows that IFNT markedly abolished in LGCs the expression of another THBS member - *THBS2*, highly expressed in these cells^9^. These findings are consistent with *THBS1*, *THBS2* and their receptors being significantly downregulated in the CL for the whole duration of the bovine pregnancy^62^.

**Figure 8:**
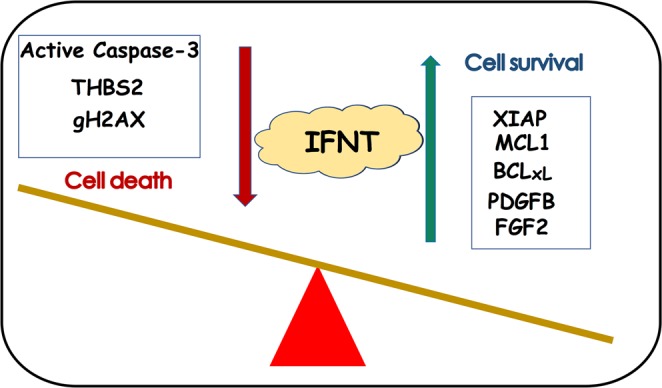
Illustrative summary depicting IFNT actions balancing life and death of bovine LGCs. IFNT elevates viable LGCs numbers, reduces apoptotic cells, and increases prosurvival proteins including XIAP, MCL1, and BCL-xL. Concurrently with its prosurvival effects, IFNT stimulates proangiogenic factors—FGF2, PDGFB. Conversely, antiapoptotic factors, active caspase-3, THBS2 and gH2AX are inhibited by IFNT. These findings portray IFNT as a prominent luteal survival factor.

We observed that FGF2 was elevated by IFNT *in vitro* (Fig. 7a) and in early pregnant CL (Fig. 8d). FGF2 plays a vital role in supporting luteal angiogenesis and cell survival *in vivo*^63^ and *in vitro*^64,65^. Besides their direct antiangiogenic, proapoptotic effects, THBS1 and THBS2 were shown to sequester FGF2 and impair its biological activities (proliferation, survival and migration)^8,9,11,66,67^. Hence, the fact that IFNT suppressed THBS2 is expected to further augment the biological functions of FGF2, whose levels were induced *in vitro* and during early pregnancy. THBS1 and THBS2 have similar expression profiles in response to PGF2a and analogous biological functions, yet THBS2 is understudied in CL and it requires future studies.

Another class of proangiogenic factors is the PDGF–PDGFR axis. In particular, PDGFB is involved in attracting pericytes to new vessels during angiogenesis, which are crucial for the stabilization and maturation of blood vessels^68^. In luteal endothelial cell network established *in vitro*, the inhibition of PDGF signaling markedly decreased their formation and sprouting^69^. Corroborating this notion, this study revealed that *PDGFB* and *PDGFAR* were stimulated by IFNT in LGCs and elevated *PDGFB* was also observed in the CL of day 18 pregnant cows.

In summary, this study establishes that IFNT directly promotes LGCs survival, it also contributes to cell health by counteracting THBSs-mediated apoptotic events. Furthermore, IFNT enhanced the production by LGCs of factors involved in luteal vasculature growth and stabilization. Previously we reported that IFNT also enhanced the survival of luteal endothelial cells and suppressed luteolytic genes in these cells and CL slices^25^. The actions of IFNT exerted on both luteal steroidogenic and endothelial cells might underline the mechanisms used by this pregnancy recognition signal in the CL to maintain its function during early pregnancy.

## Materials and Methods

### Isolation and culture of LGCs

We collected ovaries bearing large follicles (>10 mm in diameter) from a local slaughterhouse, as described previously^7,60^. Only follicles containing at least 4 million granulosa cells were included in these experiments. Granulosa cells were enzymatically dispersed by using a combination of collagenase I (5000 U), hyaluronidase III (1440 U), and deoxyribonuclease I (390 U; Sigma-Aldrich, St. Louis, MO) in Dulbecco’s modified Eagle medium (DMEM)/F-12 containing 1% l-glutamine and 1% penicillin/streptomycin (Biological Industries, Kibbutz Beit Haemek, Israel). Next, isolated granulosa cells were seeded for overnight incubation in DMEM/F-12 containing 3% fetal calf serum (FCS). The next day, media were replaced with luteinization media containing FCS (1%), insulin (2 μg/mL; Sigma-Aldrich) and forskolin (10 μM; Sigma-Aldrich). On day 6 of culture, the cells were washed with PBS and kept for 3–5 h adaptation period in DMEM/F-12 media containing1% FCS. Then cells were incubated for the times indicated in the legends either with basal media (media containing 1% FCS) or with roIFNT (0.01–10 ng/mL; a generous gift from Prof. Fuller W. Bazer, Texas A&M University) or recombinant human THBS1 (rhTHBS1; 250 ng/mL; Genentech, South San Francisco, CA) alone or with the combination of rhTHBS1 and roIFNT. At the end of the incubation period, cells were collected for either total RNA extraction or protein for protein analyses, or for flow cytometry as described below.

### Experimental procedures with animals and synchronization

The experiment was conducted in accordance with relevant guidelines and regulations at the Experimental Station Hildegard Georgina Von Pritzelwiltz, located in Londrina, PR, Brazil. The Animal Research Ethics Committee of Escola Superior de Agricultura “Luiz de Queiroz” (ESALQ)/University of São Paulo approved all procedures involving cows in this study (Protocol #2018.5.1252.11.5).

Non-lactating Bos indicus cows (n = 35) were submitted to a fixed-time AI FTAI protocol GnRH-E2 based. On the day of insemination (Day 0) cows were assigned to the following treatments: Artificial insemination group (n = 20) vs. synchronized non-inseminated group (cyclic; n = 10). Cows from the AI group were inseminated using frozen/thawed semen from two high fertility Aberdeen Angus bulls (Alta Genetics, Uberaba, Brazil). On day 17 after AI and one day before CL collection, blood samples were collected by puncture of the coccygeal vein into evacuated 10 mL tubes containing sodium heparin (Vacutainer, Dickinson, Franklin Lakes, NJ). Immediately after collection, the tubes were placed on ice and kept refrigerated until processing. Blood samples were centrifuged at 1,700 × g for 15 min and aliquots of plasma were frozen and stored in duplicates at −20 °C until assayed for progesterone. On Day 18 all cows were slaughtered and each horn was flushed using 10 mL of sterile saline solution in the AI group to assure the presence of an embryo. Pregnancy was confirmed by identification of an elongated embryo in uterine flushes and the presence of a functional CL was confirmed by measuring plasma progesterone using a commercial kit (CT Progesterone, MP Biomedicals LLC, Solon, OH, USA) following the manufacturer’s instructions. The CL were collected in cryotubes and immediately frozen in liquid nitrogen and stored at −80 °C for subsequent RNA extraction from six cows in each pregnant (P) and cyclic (C) groups.

### Western blot analyses

Proteins were extracted with a sample buffer, separated by 7.5–12.0% SDS–PAGE, and subsequently transferred to nitrocellulose membranes, as reported previously^8^. Membranes were blocked for 1 h in TBST (20 mmol/L Tris, 150 mmol/L NaCl, and 0.1% Tween 20; pH 7.6) containing 3% BSA or 5% low-fat milk, and then incubated overnight at 4 °C with the following antibodies: goat polyclonal phosphorylated STAT1 (TYR701; 1:200; Santa Cruz Biotechnology, Heidelberg, Germany); goat polyclonal STAT1 (1:200; Santa Cruz Biotechnology); rabbit anti-FGF2 antiserum (1:10 00; kindly provided by D. Schams, The Technical University of Munich), rabbit anti-MCL1 (1:1000; Cell Signaling Technology, Beverly, MA), rabbit anti-gH2AX (1:1000; Cell Signaling Technology), rabbit anti-cleaved caspase-3 (1:1000; Cell Signaling Technology), mouse anti-XIAP (1:500; Santa Cruz Biotechnology), BCL-xL (1:500; Santa Cruz Biotechnology), and rabbit anti-p44/42 total mitogen-activated protein kinase (MAPK; 1:50,000; Sigma-Aldrich). The membranes were then incubated with peroxidase-conjugated goat anti-rabbit or anti-mouse IgG (Jackson ImmunoResearch, West Grove, PA) for 1 h at room temperature. A chemiluminescent signal was generated with the SuperSignal Detection Kit for horseradish peroxidase (Thermo Fisher Scientific, Waltham, MA), and the signal was captured with ImageQuant LAS 500 (GE Healthcare Life Sciences, Marlborough, MA). The protein bands were analyzed using Gel-Pro 32 Software (Media Cybernetics, Silver Spring, MD) and the signal of anti-total MAPK (p44/42) antibody was used to correct for protein loading.

### Determination of viable cell numbers

We estimated the viable cell numbers as described previously^9^, using the XTT Kit (Biological Industries), which measures the reduction of a tetrazolium component by the mitochondria of viable cells. On the day of measurement, XTT was added to the culture media per the manufacturer’s instructions. Plates were then incubated at 37 °C for 3–5 h. Afterwards, the absorbance was read at 450 nm (reference absorbance, 630 nm).

### Analysis of cell viability/apoptosis by flow cytometry

We determined the cell viability/apoptosis using the Annexin V–FITC (PE) and PI double-staining apoptosis using the MEBCYTO Apoptosis Kit (Medical and Biological, Woburn, MA). After 48 h of incubation with 1 ng/mL of roIFNT or control media, cells were trypsinized and washed two times with PBS. Then, cells were centrifuged and suspended in 500 μL of binding buffer, after which 5 μL of Annexin V–FITC and PI staining solution were added and incubated at room temperature for 10 min. Next, the mixture was analyzed by flow cytometer (FACS) BD FACSCalibur™ (Becton Dickinson, Franklin Lakes, NJ) within 30 min. Finally, the number of apoptotic cells was defined as the sum of the Annexin V–FITC^+^/PI^−^ cells and Annexin V–FITC^+^/PI^+^ cells.

### RNA extraction and qRT-PCR

The total RNA was isolated from the CL tissue/cells using Tri Reagent (Molecular Research Center, Cincinnati, OH) per the manufacturer’s instructions. As described previously, the total RNA was reverse-transcribed and quantitative RT-PCR (qPCR) was performed using the LightCycler 96 System (Roche Diagnostics, Indianapolis, IN) with Platinum SYBR Green (SuperMix, Invitrogen, Carlsbad, CA)^25^. Table 1 lists the sequences of primers used for quantitative qPCR. All primers were designed to have single-product melting curves, as well as consistent amplification efficiencies between 1.8 and 2.2^70,71^. In addition, all amplicons were verified by sequencing. To select the most stable housekeeping gene, we applied the NormFinder algorithm; we selected *GAPDH* as a housekeeping gene, as described previously^25,71^.Furthermore, the threshold cycle number (Ct) was used to quantify the relative abundance of the gene; arbitrary units were calculated as 2^−ΔCt^ = 2^−(Ct target gene − Ct housekeeping gene)^^72^.

**Table 1:** List of primers used for qRT-PCR.

Gene name	Sequence (5′-3′)	Accession No.
*GAPDH*	f- gtcttcactaccatggagaagg r- tcatggatgaccttggccag	NM_001034034
*STAT1*	f- cagccagctcccaagtg r- gccaactcagcacctctg	NM_001077900.1
*MX2*	f-tggcaggtggaagagagc r- gagtcgatgaggtcaatgcag	NM_173941.2
*ISG15*	f- ggtatccgagctgaagcagtt r- acctccctgctgtcaaggt	NM_174366.1
*OAS1Y*	f- tttggtctggctggattacc r- taggcctggaacatcaggtc	NM_001040606.1
*FGF2*	f- tgtctccccctcactctggta r- actccctgtatagccaaaggtctg	NM_NM_174056
*PDGFRA*	f- ggcgaatcaattgtggtca r- ctcgggaaccctcagagtg	NM_001192345.3
*PDGFB*	f- ctcatagaccgcaccaatg r- cttcttcttccgcacgatc	NM_001017953.2
*THBS1*	f- atcatggctgactcaggac r- taagcccatggttccagaa	NM_174196
*THBS2*	f- gcttcgtccgctttgactac r- taggtgaggtccagggtgt	NM_176872

### Statistical analyses

All statistical analyses were conducted using GraphPad Prism version 6.01 Software (GraphPad Software, Inc., San Diego, CA). Data are presented as means ± SEM. *In vitro* experiments comprised, at least, three independent repeats; each repeat constituted cells obtained from different follicles (one follicle/cow). Data were analyzed by either Student’s *t*-test or one-way ANOVA, followed by the Bonferroni’s *post-hoc* multiple comparison test, when indicated. In this study, differences were considered as significant equal or below *P* < 0.05. Asterisks or different letters represent statistical significant differences (*P* < 0.05).
